# Implementation of an Educational Intervention for Gastric Cancer Awareness in the General Population in CELAC and Europe: A Strategy Proposed by the LEGACy Consortium

**DOI:** 10.1007/s13187-025-02578-2

**Published:** 2025-02-13

**Authors:** Juan Antonio Carbonell-Asins, Elena Jiménez-Martí, Sergio Romero, Eduardo García, Ana Miralles-Marco, Beatriz Lopez, Marisol Huerta, Carmelo Caballero, Hugo Boggino, Cinthia Gauna, Olga Beatriz Acevedo-Funes, Gabriel Benitez Nuñez, Claudia Melina Céspedes-Cardozo, Edith A. Fernandez-Figueroa, Nayeli Ortiz-Olvera, Erika Ruiz-García, Fátima Carneiro, Rita Barros, Ceu Figueiredo, Rui M. Ferreira, Tessa Suzanne Groen - van Schooten, Demi van Santvliet, Sarah Derks, Romina Luca, Maria Alsina, Arnoldo Riquelme, Andrés Cervantes, Tania Fleitas

**Affiliations:** 1 Department of Medical Oncology, Hospital Clínico Universitario, Biostatistical Unit, INCLIVA, Biomedical Research Institute, Avenida Menendez Pelayo Nro 4 Accesorio, 46010 Valencia, Spain; 2 Department of Medical Oncology Hospital Clínico Universitario, INCLIVA, Biomedical Research Institute, University of Valencia, Avenida Blasco Ibañez 17, 46010 Valencia, Spain; 3 Department of Biochemistry, University of Valencia, Avenida Blasco Ibañez XX, 46010 Valencia, Spain; 4 Vall d Hebron Institute of Oncology, Biostatistical Unit, Calle Nazaret 155-177 08035, Barcelona, Spain; 5 Genpat. Pathology Department, Guido Spano 1448, Asunción, Paraguay; 6 Oncology Department, Instituto de Previsión Social, Av Santisimo Sacramento y Dr. Manuel Peña, Asunción, Paraguay; 7 Cátedra de Biofísica, Universidad Nacional de Asunción, Campus Universitario San Lorenzo, San Lorenzo, Paraguay; 8 Núcleo B de Innovación en Medicina de Precisión, Instituto Nacional de Medicina Genómica, Periferico Sur Número 4809, Colonia Arenal, Tepepán, Alcaldia Tialpan, 14610 Ciudad de México, Mexico; 9 Departamento de Gastroenterología, UMAE, Hospital de Especialidades Dr. Bernardo Sepúlveda, Centro Médico Nacional Siglo XXI, IMSS, Avda Cuauhtemoc 330, Doctores, Cuauhtemoc, 06720 Ciudad de México, Mexico; 10 Departamento de Tumores de Tubo Digestivo, Instituto Nacional de Cancerología, Avda San Fernando 22, Belisario Dominguez, Secc 16, Tialpán, 14080 Ciudad de México, Mexico; 11 Laboratorio de Medicina Traslacional, Instituto Nacional de Cancerología, Avda San Fernando 22, Belisario Dominguez, Secc 16, Tialpán, 14080 Ciudad de México, Mexico; 12 Institute of Molecular Pathology and Immunology of the University of Porto (IPATIMUP), Rua Julio Amaral de Carvalho, 45, 4200-135 Porto, Portugal; 13 i3S–Instituto de Investigação e Inovação em Saúde, Universidade Do Porto, R. Alfredo Allen 208, 4200-135 Porto, Portugal; 14 Department of Pathology, Faculty of Medicine of the University of Porto, Alameida Prof. Hernani Monteiro, 4200-319 Porto, Portugal; 15 Department of Medical Oncology, Amsterdam UMC Location University of Amsterdam, Meibergdreef 9, Amsterdam, The Netherlands; 16 Cancer Center Amsterdam, Cancer Biology and Immunology, Meibergdreef 9, Amsterdam, The Netherlands; 17 Oncode Institute, Jaarbeursplein 6, 3521 AL Utrecht, The Netherlands; 18 Oncology Department, Instituto Alexander Flemming, Crammer 1180, C1426 Buenos Aires, Argentina; 19 Oncology Department, Vall d Hebron Institute of Oncology, Calle Nazareth 115-117, 08035 Barcelona, Spain; 20 Gastroenterology Department, Pontificia Universidad Católica de Chile, Santiago, Chile; 21 Centro Para La Prevención y Control del Cáncer (CECAN), Avenida Libertador Bernardo O`Higgins 340, Santiago de Chile, Chile; 22 CIBERONC, Instituto de Salud Carlos III, Avenida de Monforte de Lemos 5, Fuencarral-El Pardo, 28029 Madrid, Spain

**Keywords:** Gastric cancer, Gastro-esophageal cancer, Prevention, Gastric cancer risk factors

## Abstract

Gastric cancer (GC) has a poor prognosis. The LEGACy consortium has been established to enhance GC outcomes though improved primary and secondary prevention strategies. We performed an educational intervention study using an online module to disseminate knowledge about GC risk factors and symptoms to the general population. Participants were recruited through various media channels and were exposed to an online questionnaire to assess their knowledge, before and after the educational intervention. The educational intervention included an informative brochure and a short video providing essential information about GC. Primary outcome was to evaluate the overall knowledge (global score) before and after the intervention. A total of 1034 participants were evaluated before the intervention. Of those, 866 also completed the short-term and 362 the long-term questionnaire after the intervention, respectively. On a scale of 0 to 17, the baseline global score mean was 9.4 (3.2). Results showed an increase in the average global knowledge score by 1.80 (95% CI: 1.63–1.96, *p* < 0.001) and 1.81 (95% CI: 1.65–1.96, *p* < 0.001) points after completing the short and long-term questionnaires compared to the baseline respectively for all individual questions (*p* < 0.05). This interventional study showed significantly improved knowledge in most domains on GC risk factors, signs, and symptoms which could be a useful strategy for promoting cancer prevention. ClinicalTrials.gov Identifier: NCT04019808.

**Supplementary Information:**

The online version contains supplementary material available at 10.1007/s13187-025-02578-2.

## Introduction

Gastric cancer (GC) is the fifth most common cancer and the fifth most deadly cancer worldwide. The global burden of gastric cancer is over 1,000,000 new cases and 660,175 deaths per year [1]. A better understanding of the key oncogenic drivers, early detection strategies, and improved therapeutic approaches are urgently needed to improve GC outcomes. The development of GC is associated with risk factors such as *Helicobacter pylori* (HP) infection, inherited genetic predisposition, and unhealthy lifestyle habits including obesity, smoking, and consumption of alcohol and processed meat [2]. Epidemiological studies have explored the relationship between work stress and the risk of cancer, but it remains unclear on whether work stress could increase the risk of cancer [3]. Patients with GC could develop a variety of symptoms that include early satiety, postprandial fullness, abdominal pain, defecation changes, weight loss, and fatigue. Symptoms arise late and are often recognized when more than half of the tumors have already metastasized to regional lymph nodes or distant locations. In this advanced stage of the disease, systemic treatment is limited in its effectiveness [2]. Therefore, besides finding better therapies, prevention is key to improving survival. From a public health standpoint, prevention of GC could be conducted at three levels: (i) primary prevention by educating about risk factors associated with the disease, (ii) secondary prevention by early diagnosis through screening and knowledge of symptoms, and (iii) tertiary prevention by improving the diagnosis and the therapeutic approaches for advanced disease stages. Medical oncologists and other disciplines should participate in the different prevention strategies due to provide the scientific insights necessary to guarantee that such programs have a positive impact, and to disseminate information on how to prevent cancer [4].

The LEGACy consortium has been established to enhance GC outcomes through improved primary and secondary prevention strategies, involving measuring and disseminating knowledge on GC risk factors and symptoms among the general population, and promoting healthy lifestyle habits. Understanding the regional variations in biological and clinical behavior of (advanced) GC will help to create fundamentals for globally implementable diagnostic and treatment approaches. The main objective of this study is to evaluate the impact of and educational intervention about GC risk factors in European (EU) and Latin American (LATAM) countries. The secondary outcome is to study demographic and epidemiological factors related to GC knowledge [5].

## Materials and Methods

### Institutions and Partners

LEGACy is a multi-institutional research approach performed by a team of four LATAM and seven EU organizations. Centers were selected due to their excellence and expertise, capability for achieving the recruitment plan, and the commitment of each researcher involved in the project (see Annex 1).

### Study Design

The Educational intervention study consisted of an online module disseminated though various media channels, including the LEGACy project website, institutional websites affiliated with the LEGACy consortium, and Facebook and Twitter, to recruit potential participants from the general population. Initial methodology was to administer the questionnaire to participants by means of a face-to-face interview but due to the COVID pandemic outbreak we opted for an online approach. Participants provided consent through an online informed consent form. Then they completed an online questionnaire assessing their knowledge of GC risk factors and symptoms (baseline survey). After completing the questionnaire, participants received an informational brochure (https://www.legacy-h2020.eu/patients/) to read and a short video (https://www.youtube.com/watch?v=jad3ej99aeA) to visualize containing essential information about GC. After that, the same online questionnaire was completed again at short-term (immediately after the intervention) and long-term intervals (around 3 months after the intervention) to evaluate the impact of our educational intervention program.

The primary outcome was to evaluate the overall knowledge (global score) obtained before and after the intervention as a measurement of the learning curve. The secondary outcome was to study the demographic and epidemiological factors associated with the pre-intervention knowledge score that potentially influence the results.

### LEGACy Questionnaires

LEGACy questionnaires included 17 questions (Yes/No, multichoice options) focused on the knowledge of main GC risk factors and symptoms repeated three times (previously and after reading the brochure and watching the video, and after 1–3 months) as well as eight general questions which were administered during the first questionnaire (Annex 2). The questionnaires took about 15 min each. They were designed by the LEGACy Consortium guided by experts in GC prevention and carefully reviewed by the consortium partners and the European Cancer Patient Coalition organization. The most frequent GC risk factors and symptoms were selected based on the literature. Questionnaires were written in simple language and reviewed and translated into Spanish, English, Dutch, Catalan, and Portuguese by the different sites participating in this study.

### Sample Size

Assuming an unlimited population, the minimum number of subjects to include in part one (the pre-intervention survey) was 666 (with a 99% confidence level and 5% margin of error and a population proportion of 0.5).

### Statistical Analyses

Qualitative variables were described using frequencies and percentages, while quantitative variables were summarized using mean and standard deviation or median and interquartile range according to variable distribution. Normality was checked with the Shapiro–Wilk test. For quantitative variables, the mean comparison was carried out using Student’s *t*-test if there is normality; otherwise, the Mann–Whitney test was used. For qualitative variables, comparison of percentages between groups was studied using Fisher’s exact test for dichotomous variables or chi-square test for contingency tables with more than two categories.

To evaluate the impact of intervention, a global score to summarize GC knowledge was constructed by giving one point for each correctly answered question. As the total number of questions is 17, the maximum global score that can be achieved is 17 points if the interviewer responds correctly to all of them.

*p*-values below 0.05 are considered significant. The primary outcome was evaluated using a linear mixed model with global score as dependent variable, intervention as independent factor, and respondent identifier as random effect. The secondary outcome was studied using linear regression model with global score as dependent variable and demographic factors as independent variables. Akaike’s information criterion was used to select variables in a backward stepwise procedure. Variables included in the full model were age, sex (male or female), civil status (single, married, other, or no answer), education level (no studies, primary, secondary, or tertiary), country of residence (Argentina, Mexico, Paraguay, Portugal, Spain, or other), general health (excellent, good, average, bad, very bad, or no answer), oncological disease (no, yes, do not know, or other), and type of disease (other, GC, or no answer). Software used for all analysis is R in its 4.0.2 version [6]. The cut-off for test significance was set to *p* < 0.05. All tests were two-sided.

## Results

Data from the on-line form was collected and curated and any personal identification data was eliminated. Incomplete data and duplicates were also eliminated. At baseline (pre-intervention), a total of 1034 participants were evaluated after completing the online questionnaire about their knowledge on GC risk factors and symptoms. Of those, 866 completed the survey immediately after the intervention (short-term), and 362 answered the survey three months after (long-term). The primary outcome was evaluated in 866 participants for the short-term intervention effect and 362 for the long-term intervention effect, while the secondary outcome was studied in 1034 participants (Table 1). Data are available on Carbonell, et al. (2024), “LEGACy CS3”, Mendeley Data, V1, 10.17632/v88ytm9jrf.1.

**Table 1:** Main demographic characteristics of the population participating in the study

Characteristics		All participants (*N* = 1034)
Age (median, IQR)		28 (21–45)
Sex (*n*, %)	Male	388 (37.6)
	Female	644 (62.4)
Civil status (*n*, %)	Single	621 (60.1)
	Married	306 (29.6)
	Other	87 (8.4)
	NR/DK	20 (1.9)
Educational level (*n*, %)	None	22 (2.1)
	Primary	27 (2.6)
	Secondary	169 (16.3)
	Tertiary	816 (78.9)
Country of residence (*n*, %)	Argentina	12 (1.2)
	Mexico	163 (15.8)
	Other	25 (2.4)
	Paraguay	544 (52.6)
	Portugal	116 (11.2)
	Spain	174 (16.8)
Overall self-assessed health status (*n*, %)	Excellent	125 (12.1)
	Good	526 (50.9)
	Moderate	329 (31.8)
	Bad	37 (3.6)
	Very bad	9 (0.9)
	NR/DK	8 (0.8)
Family or previous history of cancer? (*n*, %)	No	368 (35.6)
	Yes	494 (47.8)
	NR/DK	172 (16.6)
Type of cancer (*n*, %)	None	306 (29.6)
	GC	54 (5.2)
	Other	674 (65.2)
Baseline score (mean, standard deviation)	Mean (SD)	9.4 (3.2)

GC risk factors, symptoms, and prevention knowledge

*NR/DK* no answer/no response, *IQR* interquartile range

At baseline, the median age of participants was 28 (range 21–45), and 644 (62.3%) were females. The majority of participant resided in Paraguay (*n* = 544;52.6%), followed by Spain (*n* = 174; 16.8%), and Mexico (*n* = 163; 15.8%). The baseline global knowledge score for the population participating in this study was 9.4 (standard deviation 3.2; Table 1). Additional demographic data are also presented in Table 1.

**Fig. 1:**
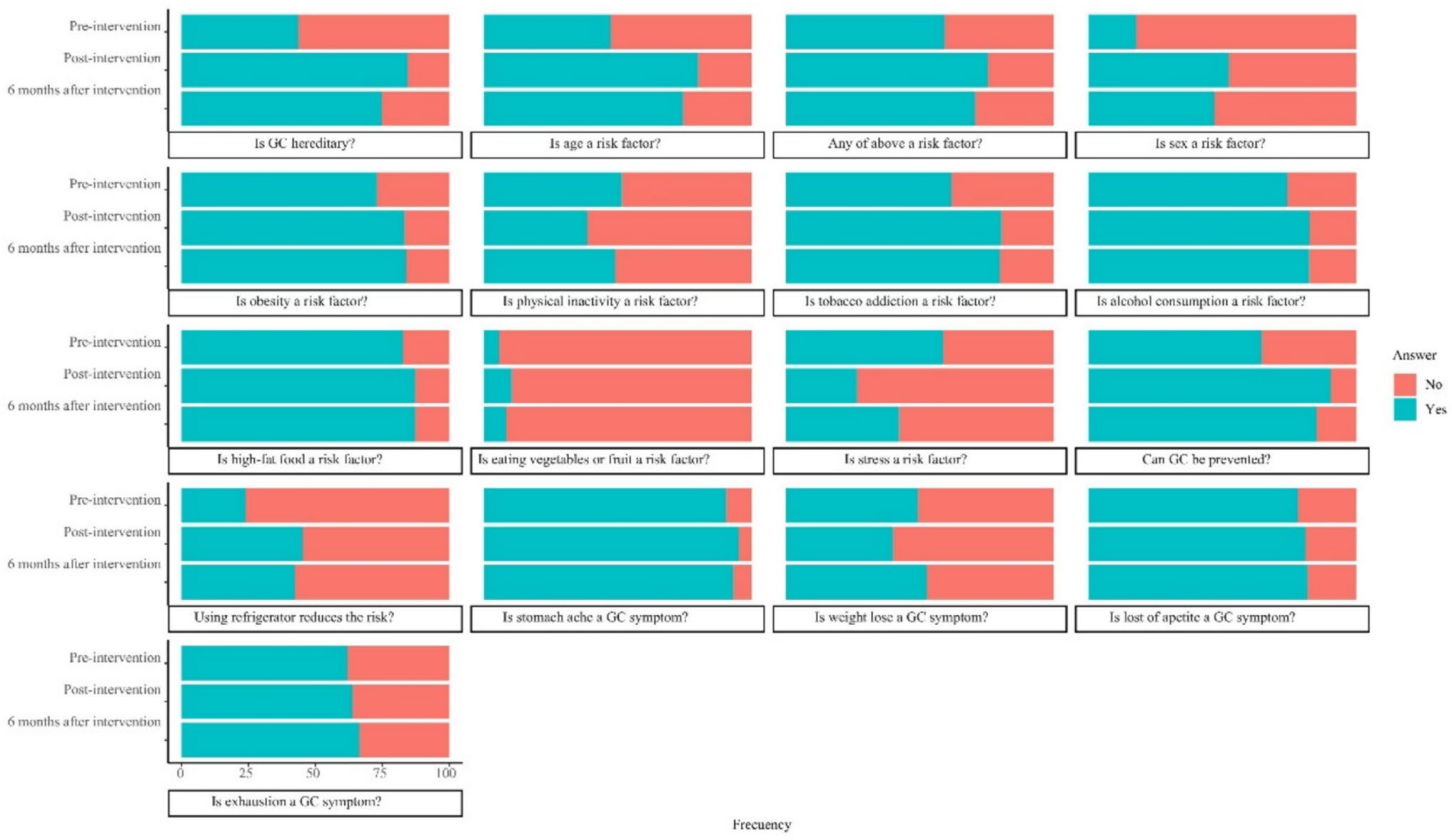
Gastric cancer risk factors, symptoms, and prevention knowledge pre-intervention, short term, and long term after intervention

A set of 17 questions was given to participants at three different time points (pre-intervention, immediately post-intervention (short term), and around 3 months after intervention (long term)), in order to evaluate knowledge about GC risk factors, symptoms, and prevention of the general population. Figure 1 depicts the percentage of questions answered correctly and incorrectly at each time point for each question. General estimation equations were used to evaluate changes in response between pre-intervention, short term and long-term responses respectively after intervention independently in each of the 17 questions. The results indicate that the intervention was effective in improving knowledge for all individual questions (*p* < 0.05).

### Impact of Educational Intervention Program

A linear mixed model was used to evaluate the efficacy of the educational intervention program. We found that after completion of the second questionnaire, there was an increase in the average global knowledge score by 1.80 points (95% CI: 1.63–1.96, *p* < 0.001). Similarly, completion of the third questionnaire resulted in a higher average global knowledge score, with an increase of 1.81 points (95% CI: 1.65–1.96, *p* < 0.001) compared to the first questionnaire.

### Demographic and Epidemiological Determinants of Gastric Cancer Knowledge

Using linear regression, we studied demographic and epidemiological factors related to GC knowledge at baseline. According to Akaike’s Information Criterion, the best model shows that age was negatively associated with global knowledge scores, meaning that older participants had worse scores (*p* = 0.013). The educational level was also associated with knowledge scores, and participants with higher levels of education had an average higher global score. Finally, there were differences between countries, with participants from Spain or Portugal showing higher baseline global knowledge scores compared to participants from Argentina (*p* = 0.028 and *p* = 0.002, respectively, Table 2).

**Table 2:** Univariable and multivariable linear regression for GC baseline knowledge

		Univariable	Multivariable
Age	[12.0, 83.0]	0.01 (0.00 to 0.03, *p* = 0.029)	− 0.02 (− 0.04 to − 0.00, *p* = 0.013)
Sex	Male	*Reference*	–
	Female	0.35 (− 0.05 to 0.75, *p* = 0.084)	–
Civil status	Single	*Reference*	–
	Married	0.79 (0.36 to 1.23, *p* < 0.001)	–
	Other	0.48 (− 0.23 to 1.19, *p* = 0.182)	–
	No answer	− 0.87 (− 2.28 to 0.53, *p* = 0.223)	–
Education level	No studies	*Reference*	*Reference*
	Primary	2.76 (0.99 to 4.53, *p* = 0.002)	2.04 (0.29 to 3.79, *p* = 0.023)
	Secondary	2.26 (0.86 to 3.66, *p* = 0.002)	1.87 (0.50 to 3.23, *p* = 0.007)
	Tertiary	2.92 (1.59 to 4.26, *p* < 0.001)	2.48 (1.18 to 3.78, *p* < 0.001)
Country of residence	Argentina	*Reference*	*Reference*
	Mexico	1.88 (0.06 to 3.69, *p* = 0.043)	2.00 (0.18 to 3.82, *p* = 0.031)
	Other	1.96 (− 0.17 to 4.09, *p* = 0.071)	1.68 (− 0.44 to 3.80, *p* = 0.121)
	Paraguay	0.81 (− 0.97 to 2.58, *p* = 0.373)	0.48 (− 1.28 to 2.24, *p* = 0.596)
	Portugal	2.80 (0.96 to 4.64, *p* = 0.003)	2.97 (1.11 to 4.82, *p* = 0.002)
	Spain	2.09 (0.28 to 3.90, *p* = 0.024)	2.02 (0.21 to 3.83, *p* = 0.028)
General health	Excellent	*Reference*	–
	Good	0.28 (− 0.34 to 0.90, *p* = 0.378)	–
	Average	0.48 (− 0.18 to 1.13, *p* = 0.153)	–
	Bad	0.62 (− 0.54 to 1.79, *p* = 0.296)	–
	Very bad	− 1.36 (− 3.51 to 0.79, *p* = 0.215)	–
	No answer	− 1.28 (− 3.70 to 1.14, p = 0.299)	–
Oncological disease	No	*Reference*	–
	Yes	0.72 (0.29 to 1.15, *p* = 0.001)	–
	Do not know	− 0.60 (− 1.31 to 0.10, *p* = 0.094)	–
	No answer	0.10 (− 0.68 to 0.89, *p* = 0.794)	–
Type of disease	Other	*Reference*	–
	GC	− 0.36 (− 1.27 to 0.56, *p* = 0.449)	− 0.59 (− 1.48 to 0.30, *p* = 0.194)
	No answer	− 0.25 (− 0.68 to 0.18, *p* = 0.248)	− 0.64 (− 1.11 to − 0.18, *p* = 0.007)

## Discussion

When diagnosed in its early stages, GC has a 5-year survival rate of 60%. This generally occurs in countries where screening strategies and HP eradication are part of the National Health plans, such as Japan [7]. On the other hand, the high mortality and lower survival rates in western populations can be attributed to the lack of screening strategies, the late appearance of symptoms, and the absence of awareness campaigns [1].

Educational intervention strategies have shown impact in early detection in other tumor types such as breast, colorectal, and cervical cancer [8–10]. Recently, 12 recommendations were proposed for GC prevention in the Americas based on the best evidence available including the following: (1) strengthen population-based cancer registries; (2) support development and dissemination of standards for quality care; (3) enable training of health care workforce; (4) establish HP management registration; (5) establish a surveillance system of HP antibiotic resistance; (6) assure key considerations for HP treatment; (7) assure endoscopic surveillance of patients with high-risk gastric premalignant conditions; (8) establish key interventions directed to hereditary factors and GC families; (9) conduct endoscopic campaigns in high-risk populations, particularly those residing in rural areas; (10) strengthen smoking regulations; (11) strengthen strategies to reduce salt (sodium) intake; and (12) establish community education programmed [11].

The impact of educational intervention strategies can be evaluated based on four levels according to Kirkpatrick proposal [12], including perception, knowledge, behavior, and the organizational level. Our educational intervention contributes to the first two levels, perceptions and knowledge related to GC awareness.

Overall, prior to the educational intervention, the level of knowledge about GC risk factors and symptoms in our study population was insufficient, as the mean baseline score was 9.4 out of a total of 17 points (55.3%). Our study intervention resulted in significantly improved knowledge in all domains of GC risk factors, signs, and symptoms. The average global knowledge score increased by 1.80 points (95% CI: 1.63–1.96, *p* < 0.001) and by 1.81 points (95% CI: 1.65–1.96, p < 0.001) after the second and third questionnaires, respectively, compared to the first one performed before the intervention. This suggests that the main messages were retained over time. Moreover, such strategies should be given in simple wording and adapted to the language of the respective countries, and this has been shown as a cost-effective method that impacts cancer control [13].

However, there are some limitations to be considered. In our study, not all the study populations completed the second and third questionnaires. Biases, such as the level of education, age, country, and gender, also need to be considered. The questionnaire was designed by the LEGACy Consortium and reviewed by a patient association. Moreover, two villages in Spain (Aras de los Olmos, Corbera) were used as pretest and pilot but no further validation was done.

We have created an online educational intervention that, although valuable, has its limitations. Effective monitoring and engaging participants present challenges and there is a potential bias favoring those with higher digital literacy. However, this approach has yielded valuable insights for developing effective strategies to enhance knowledge and awareness of GC.

Structured, interactive patient education programs had shown superior impact than lecture-based provision of information in regard to short-term and long-term knowledge as well as short-term coping and QoL for gastric cancer [14]. Aligned with this idea, and based on the results of our study, we recommend exploring similar interventions conducted in person, specifically targeting small groups of individuals with specific key messages according to the age and risk of the respective target groups.

## Supplementary Information

Below is the link to the electronic supplementary material.

Supplementary file1 (DOCX 28 KB)

## Data Availability

The data sets (deidentifying participant data and data dictionary) used and/or analyzed during the current study are available in Carbonell, et al. (2024), “LEGACy CS3”, Mendeley Data, V1, 10.17632/v88ytm9jrf.1. The study protocol was published in [5].

## Declarations

### Clinical Trial

The clinical trial described in this paper was registered at Clinicaltrials.gov under the registration number NCT04019808 (Legacy 3) on July 15th, 2019.
