# Supplementary material for: Implementation of an Educational Intervention for Gastric Cancer Awareness in the General Population in CELAC and Europe: A Strategy Proposed by the LEGACy Consortium
Source: J Cancer Educ. 2025 Feb 13;40(5):804–11. doi: 10.1007/s13187-025-02578-2 (PMC12504374; doi:10.1007/s13187-025-02578-2)
Supplement: Supplementary file 1 — Supplementary file1 (DOCX 28 KB) [file 13187_2025_2578_MOESM1_ESM.docx]

**Figures, tables and additional files**

### **Annex 1: Study sites and PI/ coordinators**

| **Site** | **Address** | **Pincipal investigator/ coordinators** | **Type of Center** |
| --- | --- | --- | --- |
| Amsterdam UMC, Department of Medical Oncology, Cancer Center Amsterdam, Amsterdam, The Netherlands and Oncode Institute | De boelelaan, 1117  1081 HV Amsterdam, The Netherlands | Sarah Derks | Hospital University Center |
| Instituto Investigación Sanitaria INCLIVA (INCLIVA), Medical Oncology Department, Hospital Clínico Universitario de Valencia | Avenida Blasco Ibañez, 17, 46010, Valencia, Spain | Tania Fleitas (Scientific coordinator) | Hospital University Center |
|  |  | Andrés Cervantes (PI) |  |
| Institute of Pathology and Immunology of University of Porto. Department of Anatomic Pathology, Centro Hospitalar São João | Alameda Prof. Hernâni Monteiro  4200-319 Porto, Portugal | Fatima Carneiro | Hospital University Center |
| Instituto Nacional de Cancerología (INCAN), Translational Medicine Laboratory & GI Cancer Department | San Fernando N.22, Colonia Seccion XVI,  14080, Mexico City, Mexico | Erika Ruiz | Hospital University Center |
| Vall d’Hebron Institute of Oncology (VHIO), Medical Oncology Department | Vall d’Hebron University Hospital  Centro Cellex, Calle Natzaret, 115-117  08035 Barcelona, Spain | Marc Diez | Hospital University Center |
| Departamento de hematologia oncologia, facultad de medicina, pontificia universidad catolica de Chile | Diagonal Paraguay 362, 8330077, Santiago, Chile | Arnoldo Riquelme | Hospital University Center |
|  |  | Garreth Owen |  |
| GenPat | Guido Spano 1448 e/ Ohiggins y Dr. Morra, Asunción, Paraguay | Carmelo Caballero | Hospital University Center |
| Instituto Alexander Fleming, Medical Oncology Department | Avenida Crámer, 1180 C1426ANZ Buenos Aires, Argentina | Juan O`Connor | Hospital University Center |

**Annex 2: On-line form**

**INTRODUCTION**

LEGACy study | Demographic questionnaire 1

“Interventional study at the primary and secondary levels of prevention for knowledge regarding Gastric Cancer risk factors in European and Latin American populations"

We appreciate your participation. First of all we will provide you the most relevant information about your participation in LEGACy study and about the processing of your personal data. In a second section you will find the questions of the questionnaire.

**SECTION 1. PARTICIPANT INFORMATION**

Below you will find information about the LEGACY study and your participation in it. If you would like to obtain more information or ask additional questions to the information included here, please contact to legacy@incliva.es

**1. Voluntary Participation**

You have been invited to participate in the study because we are interested in the degree of awareness across the general population of the risk factors associated with gastric cancer.

You should know that your participation in this study is voluntary and that you can decide NOT to participate. If you decide to participate, you may still change your mind and withdraw your consent at any time.

By agreeing to participate in this study you consent for us to access and process some of your personal data in the way described below.

**2. Study Objective and Justification**

This study is part of the strategy of the project: "European and Latin American Consortium for the Management of Gastric Cancer through Personalized Medicine" LEGACY, which aims to improve the Gastric Cancer prognosis in Europe and Latin America. This is an international study that will be carried out in European and Latin American countries (Spain, Argentina, Holland, Paraguay, Germany, Mexico, Belgium, Chile and Portugal).

The main aim of this study is to determine awareness levels in European and Latin American populations of the risk factors and warning signs in the general population associated with gastric cancer.

**3. Study Description and Activities**

Your contribution to the study will consist of responding to a survey where you will be asked questions about your knowledge of the risk factors and warning signs of gastric cancer.

If you accept to participate in the study you will have to answer an online questionnaire on three times and each questionnaire will take approximately 15 minutes. The procedure to be followed is as follows:

1. Completion of Questionnaire 1 (this questionnaire). The aim of this questionnaire is to find out your current knowledge about the risk factors that are related to gastric cancer. We will ask you to watch a video and read an information leaflet, which will explain important aspects of gastric cancer and its symptoms and finally confirm the degree of new knowledge you have acquired about this disease.

2. Completion of Questionnaire 2, Three months after completion of the first questionnaire, you will receive an alert on your mobile phone or e-mail asking you to complete the questionnaire again. This will help us to assess the long-term educational intervention.

**4. Relevant Information**

The highly valuable data collected will be integrated into the project's centralised database for subsequent analysis by the entire research team. The conclusions will be published and disseminated to the scientific community and the general public.

It is quite likely that you will not see any benefit to your health from participating in this study, but it may help to improve educational campaigns on the risk factors and warning signs of gastric cancer that will help towards earlier disease diagnosis.

**5. Personal data process information.**

If you accept to participate in the study, the personal data you provide will be processed in compliance with European Regulation 2016/679 on Data Protection. Following we will provide you the basic information about the treatment and at the end of the questionnaire you will receive the complete information in the e-mail you will provide.

Who is the person responsible for your data processing? The institutions that make up the LEGACY project consortium:

- IIS INCLIVA - Spain - www.incliva.es
- IPATIMUP - Portugal - www.ipatimup.pt/Site/
- GENPAT - Paraguay - genpat.lab@gmail.com
- VHIO - Spain - www.vhio.net/en/
- VUMC - Netherlands - www.vumc.com
- PUC - Chile - www.uc.cl
- IAF - Argentina - www.alexanderfleming.org/es/
- INCAN - Mexico - www.incan-mexico.org/incan/incan.jsp

Purposes of data processing: The data will be processed to achieve the objective of the LEGACY study (section 2)

Legal basis for the processing: Your consent on this form.

Recipients and communications: Your personal data will NOT be shared or sent to recipients outside the LEGACY Project consortium

International transfers: It is not foreseen that personal data will be processed outside the European territory

Retention period: Your personal data will be kept for the time necessary to achieve the objectives of the LEGACY Project and to comply with current regulations.

You may request to exercise your rights regarding this processing of personal data at: privacy.legacy@incliva.es

If you would like more information about this project, please click on the following link: https://legacy-h2020.eu/

**SECTION 2. PARTICIPANT'S CONSENT**

In order to continue with the questionnaire, you need to confirm all of the following options.

It is necessary in order to comply with the legislation and regulations for conducting clinical studies and processing personal data.

Choose as many options as you wish

1. I have read the information regarding the study
2. I consider the information received is sufficient.
3. I understand that my participation is voluntary
4. I freely agree to participate in the study
5. I consent to the processing of my personal data for this research
6. I understand that I am free to withdraw from the study, without having to give any explanation.
7. At the end of the questionnaire, I will receive this information in my e-mail.

**SECTION 3. GASTRIC CANCER KNOWLEDGE SURVEY (1^st^ and 2^nd^ QUESTIONNAIRE)**

**FIRST PART**

Interview Date: DD/MM/YYYY

**I. GC knowledge**

- Do you know if the stomach cancer can be inherited? (Yes / No / I don´t know)

**II. GC risk factors knowledge**

Select from the following factors, which you consider may be related to stomach cancer.

1. Age (Yes / No)
2. Gender (Yes / No)
3. Obesity or overweight (Yes / No)
4. Physical inactivity (Yes / No)
5. Tobacco addiction (Yes / No)
6. Alcohol intake (Yes / No)
7. Diet with salty, fried, spicy, sausage, high fat foods (Yes / No)
8. Consumption of vegetables or fruits (Yes / No)
9. Stress (Yes / No)
10. Having a family member with a stomach cancer diagnosis (Yes / No)

**III. GC Symptoms**

Select the symptoms, that may be related to Gastric Cancer?

1. Stomach discomfort (Yes / No)
2. Weight loss (Yes / No)
3. Loss of appetite (Yes / No)
4. Exhaustion (Yes / No)

**IV. GC Prevention knowledge**

- Do you know that the stomach cancer can be prevented? (Yes / No / I don´t know)
- Do you know that the use of the refrigerator to preserve food lowers the risk of stomach cancer? (Yes / No / I don´t know)

**V. General Information**

1. Age (number)
2. Sex (Male / Female / Other)
3. Civil status (Single / Married / Other / Prefer not to answer)
4. Educational level (Primary / Secondary / Higher / No official education / Prefer not to answer)
5. Country of Birth
6. Country of residence
7. How would you rate your overall health? (Excellent / Good / Moderate / Bad / Very bad / Prefer not to answer)
8. Have you or any family member suffered any cancer disease? (Yes / No / I don´t know / Prefer not to answer)
9. If yes to question 8, then: What type of cancer disease? (Gastric cancer / Other)

**SECOND PART**

- Did you watch the video? (Yes / No)
- Did you read the brochure? (Yes / No)

**I. GC knowledge**

- Do you know if the stomach cancer can be inherited? (Yes / No / I don´t know)

**II. GC risk factors knowledge**

Select from the following factors, which you consider may be related to stomach cancer.

1. Age (Yes / No)
2. Gender (Yes / No)
3. Obesity or overweight (Yes / No)
4. Physical inactivity (Yes / No)
5. Tobacco addiction (Yes / No)
6. Alcohol intake (Yes / No)
7. Diet with salty, fried, spicy, sausage, high fat foods (Yes / No)
8. Consumption of vegetables or fruits (Yes / No)
9. Stress (Yes / No)
10. Having a family member with a stomach cancer diagnosis (Yes / No)

**III. GC Symptoms**

Select the symptoms, that may be related to Gastric Cancer?

1. Stomach discomfort (Yes / No)
2. Weight loss (Yes / No)
3. Loss of appetite (Yes / No)
4. Exhaustion (Yes / No)

**IV. GC Prevention knowledge**

- Do you know that the stomach cancer can be prevented? (Yes / No / I don´t know)
- Do you know that the use of the refrigerator to preserve food lowers the risk of stomach cancer? (Yes / No / I don´t know)

**SECTION 4. GASTRIC CANCER KNOWLEDGE SURVEY (3^rd^ QUESTIONNAIRE)**

Interview Date: DD/MM/YYYY

**I. GC knowledge**

- Do you know if the stomach cancer can be inherited? (Yes / No / I don´t know)

**II. GC risk factors knowledge**

Select from the following factors, which you consider may be related to stomach cancer.

1. Age (Yes / No)
2. Gender (Yes / No)
3. Obesity or overweight (Yes / No)
4. Physical inactivity (Yes / No)
5. Tobacco addiction (Yes / No)
6. Alcohol intake (Yes / No)
7. Diet with salty, fried, spicy, sausage, high fat foods (Yes / No)
8. Consumption of vegetables or fruits (Yes / No)
9. Stress (Yes / No)
10. Having a family member with a stomach cancer diagnosis (Yes / No)

**III. GC Symptoms**

Select the symptoms, that may be related to Gastric Cancer?

1. Stomach discomfort (Yes / No)
2. Weight loss (Yes / No)
3. Loss of appetite (Yes / No)
4. Exhaustion (Yes / No)

**IV. GC Prevention knowledge**

- Do you know that the stomach cancer can be prevented? (Yes / No / I don´t know)
- Do you know that the use of the refrigerator to preserve food lowers the risk of stomach cancer? (Yes / No / I don´t know)
